# PR Interval as a Valuable Predictor of Tilt Table Test Results in Patients With Neurally Mediated Syncope

**DOI:** 10.1111/anec.70054

**Published:** 2025-01-31

**Authors:** Mohammad Hossein Nikoo, Roozbeh Narimani‐Javid, Alireza Kamrava, Sasan Shafiei, Salma Nozhat, Hosein Fatemian, Ali Asadzadeh, Mehdi Motahari Moadab, Fatemeh Ghanbari, Alireza Arzhangzadeh

**Affiliations:** ^1^ Non‐Communicable Disease Research Centre Shiraz University of Medical Sciences Shiraz Iran; ^2^ Research Center for Advanced Technologies in Cardiovascular Medicine, Cardiovascular Diseases Research Institute Tehran University of Medical Sciences Tehran Iran; ^3^ Student Research Committee Shiraz University of Medical Sciences Shiraz Iran; ^4^ Department of Cardiology, School of Medicine Shiraz University of Medical Sciences Shiraz Iran

**Keywords:** ambulatory electrocardiographic monitoring, neurally mediated syncope, PR interval, tilt‐table test, vasovagal syncope

## Abstract

**Background:**

Neurally mediated syncope (NMS) is the primary cause of temporary and self‐limiting loss of consciousness. The tilt table test (TTT) has been consistently employed as a supplementary diagnostic tool for syncope evaluation. However, TTT requires specialized equipment, which is lacking in several emergency room and clinic environments. We hypothesized that patients susceptible to NMS may have higher parasympathetic tone. Thus, this study investigates the correlation between PR interval and Herat rate variability parameters as indicators of parasympathetic tone and TTT results.

**Methods:**

We included 213 patients referred to our cardiology clinic with an impression of NMS in 2022 and 2023. Data was retrospectively collected from 24‐h ambulatory electrocardiographic monitoring recordings, TTT results, and patients' history and physical examination records.

**Results:**

The analysis of the PR interval revealed a mean duration of 155 ms (95% CI: 148.61, 161.39) in negative TTT patients and 164.21 ms (95% CI: 158.44, 169.97) in positive TTT patients, indicating a statistically significant difference between two groups (*p* = 0.035). We also found that patients with a PR interval duration exceeding 160 ms demonstrated a significantly higher prevalence of positive TTT compared to those with a PR interval duration of less than 160 ms (*p* < 0.001, OR: 3.911, 95% CI: 2.143, 7.140).

**Conclusions:**

Our study suggests a PR interval longer than 160 milliseconds as a valuable tool for predicting TTT results and identifying patients at higher risk of NMS.

## Graphical abstract

A longer PR interval correlates with a positive tilt table test. This suggests that an AEM‐derived PR interval duration longer than 160 ms may be a valuable tool for predicting TTT results and identifying patients at higher risk of NMS.

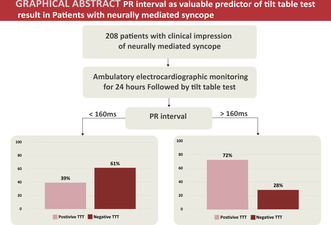

## 1 Introduction

Neurally mediated syncope (NMS) is the primary cause of temporary and self‐limiting loss of consciousness. It occurs when there is insufficient blood flow to the brain due to reflex vasodilation, which can be along with cardioinhibition (Krediet et al. 2002). The occurrence of syncope is highly prevalent among the general population, particularly Young and female individuals, with an estimated cumulative incidence of the initial syncope episode reaching around 10% over 80 years (Brignole and Hamdan 2012; Task Force for the Diagnosis and Management of Syncope; European Society of Cardiology (ESC); European Heart Rhythm Association (EHRA); Heart Failure Association (HFA); Heart Rhythm Society (HRS) et al. 2009; Soteriades et al. 2002; Sun, Emond, and Camargo Jr. 2004). The prognosis of individuals experiencing syncope is generally influenced by the severity of the underlying disease instead of the syncope episode itself. The overall prognosis is typically favorable when structural or electrical heart disease is not present (Sun, Emond, and Camargo Jr. 2004). However, it is important to note that there is still the possibility of syncope recurrences and physical damage (Brignole et al. 2009).

NMS arises due to the activation of receptors regulated by the autonomic nervous system (ANS) (White and Tsikouris 2000). Under the condition of maintaining an upright posture, the blood volume undergoes a downward redistribution as a result of gravitational forces. Within 2–3 min, an estimated 10% of the total blood volume is quickly displaced in the splanchnic and pelvic organs and lower extremities. As the duration of standing increases, this displacement is exacerbated due to transcapillary diffusion of blood (Smith, Porth, and Erickson 1994). Cardiac C fibers exhibit a response to the reduction in preload and ventricular volume and the following elevated sympathetic tone due to hypovolemia. The consequent activation of the parasympathetic nervous system leads to reflex bradycardia and syncope development. This response is particularly evident in young adults with ANS lability (Zaqqa and Massumi 2000).

The initial steps in evaluating patients presenting with syncope include a complete history and physical examination followed by a 12‐lead Electrocardiogram (ECG) (Brignole 2007). The tilt table test (TTT) has also been employed as a supplementary diagnostic tool for syncope. It is a valuable tool in the clinical assessment of cardiovascular autonomic function, as it allows for a simulation of syncope within a controlled clinical environment. TTT remains recommended for the evaluation of patients with transient loss of consciousness, according to the guidelines of the American College of Cardiology/American Heart Association/Heart Rhythm Society (ACC/AHA/HRS) and the European Society of Cardiology (ESC) (Brignole et al. 2018; Shen et al. 2017a). Studies have shown that a positive TTT has high levels of specificity ranging from 92% to 94% for diagnosing NMS (Task Force for the Diagnosis and Management of Syncope; European Society of Cardiology (ESC); European Heart Rhythm Association (EHRA); Heart Failure Association (HFA); Heart Rhythm Society (HRS) et al. 2009; Simova 2015).

Ambulatory electrocardiographic monitoring (AEM) is another beneficial tool in cases of unexplained syncope or when there is a suspicion of arrhythmia based on the patient's medical history, particularly in patients at a higher risk of experiencing arrhythmias (Subbiah et al. 2008). It helps detect cardiac‐related causes of syncope, including arrhythmias. The PR interval on the electrocardiogram indicates the flow of electrical signals through the atria and the atrioventricular (AV) node (Goldman and Schafer 2016). Variations in the duration of the PR interval may arise due to changes in autonomic neural tone or fibrosis within the various anatomical components of the heart or as a consequence of pacemaker displacement toward the AV node. It has been shown that the ANS significantly influences the PR interval. The activation of the sympathetic nervous system leads to a reduction in the PR interval, while activating the parasympathetic nervous system increases the PR interval (Alboni et al. 1986; Pirola and Potter 1990). The heart rate variability (HRV) parameters reported by the AEM, including SDNN (Standard deviation of NN intervals), SDANN (Standard deviation of the average NN intervals), SDNN index (Mean of the standard deviations of all the NN intervals for each 5 min segment), rMSSD (Root mean square of successive differences between normal heartbeats), and pNN50 (Percentage of successive RR intervals that differ by more than 50 ms) are also indicators of ANS activity (Shaffer and Ginsberg 2017).

This study aimed to introduce novel and accessible predictors of TTT results and consequent diagnosis of NMS. We hypothesized that patients susceptible to NMS may have higher parasympathetic tone. Thus, this study investigates the correlation between PR interval and HRV parameters as indicators of parasympathetic tone and TTT results.

## 2 Methods and Materials

### 2.1 Study Population

We retrospectively reviewed the recordings of all patients with complaints of syncope who were referred to an outpatient cardiology clinic affiliated with Shiraz University of Medical Sciences between February 2022 and October 2023. Patients with syncope who were considered to have NMS on initial assessment and normal neurological and cardiovascular examinations without evidence of pre‐excitation, long and short QT syndromes, or Brugada syndrome in 12‐lead ECG were enrolled in this study. Patients with a Schwartz score of less than two or no data for AEM and TTT were excluded. We reviewed the records of 385 patients with TTT results, of which 208 had AEM record data.

### 2.2 24‐Hour Ambulatory Electrocardiographic Monitoring

All patients were monitored for 24 h by an Ambulatory Electrocardiographic system (DMS 300‐4A recorders, Oxford Instruments, UK) an average of 10 days before TTT. The AEM was performed on an outpatient basis, and its recordings were transferred to a computer program (CardioScan II, DM Software Inc., USA) and analyzed. Before analyzing the data, recordings were preprocessed, and artifacts were excluded from analysis with a visual examination of the recordings by a cardiologist. The PR interval duration was calculated as the average of PR interval records collected over the 24‐h AEM. The lowest and highest heart rate and time domain HRV parameters, including RMSSD, pNN50, SDNN, SDANN, and SDNN index, were also calculated in accordance with the previous guidelines published by the European Society of Cardiology (Task Force of the European Society of Cardiology and the North American Society of Pacing and Electrophysiology 1996).

### 2.3 Tilt Table Test

Before tilt table testing, patients stayed in the supine position for at least 5 min. Then, patients were wrapped to avoid falls and traumatic injuries. Blood pressure and ECG monitoring were done during the test. The TTT was performed according to the Italian protocol (Bartoletti et al. 2000). The passive phase lasts 20–45 min during the TTT. Later, 0.4 mg nitroglycerine was given sublingually to increase the sensitivity of the test. Patients' positions were changed to an upright position for up to 15 min. The tilt table was terminated at syncope or after protocol termination.

### 2.4 Statistical Methods

All statistical analyses were performed using SPSS version 26.0 for Windows (Statistical Package for Social Sciences, SPSS Inc., Chicago, IL, USA). We used the Chi‐square test to compare two quantitative variables and the independent *t*‐test to compare quantitative and qualitative variables. Pearson correlation was also used to assess the correlation between two quantitative variables.

## 3 Results

A total of 208 participants were retrospectively included in the current study, comprising 89 males (43.2%) and 119 females (56.8%), with an average age of 43.51 years (±18.99). There was a diverse age range among participants from 5 to 87 years. One hundred eight patients tested positive for the TTT (50.7%), while 100 tested negative (46.9%), with five missing results (2.3%). The analysis of the PR interval revealed a mean of 155 ms (95% CI: 148.61, 161.39) for negative tilt test patients and 164.21 ms (95% CI: 158.44, 169.97) for positive TTT patients. We found a statistically significant difference in the mean PR interval between the two groups (*p* = 0.035; Table 1).

**TABLE 1:** Demographic characteristics.

Parameter	*N* (%)
Age	43.51 ± 18.99
Male	92 (44.2)
Tilt table test	
Positive	108 (52)
Negative	100 (48)
PR Interval	
< 160 ms	126 (60.57)
> 160 ms	82 (39. 42)

**FIGURE 1:**
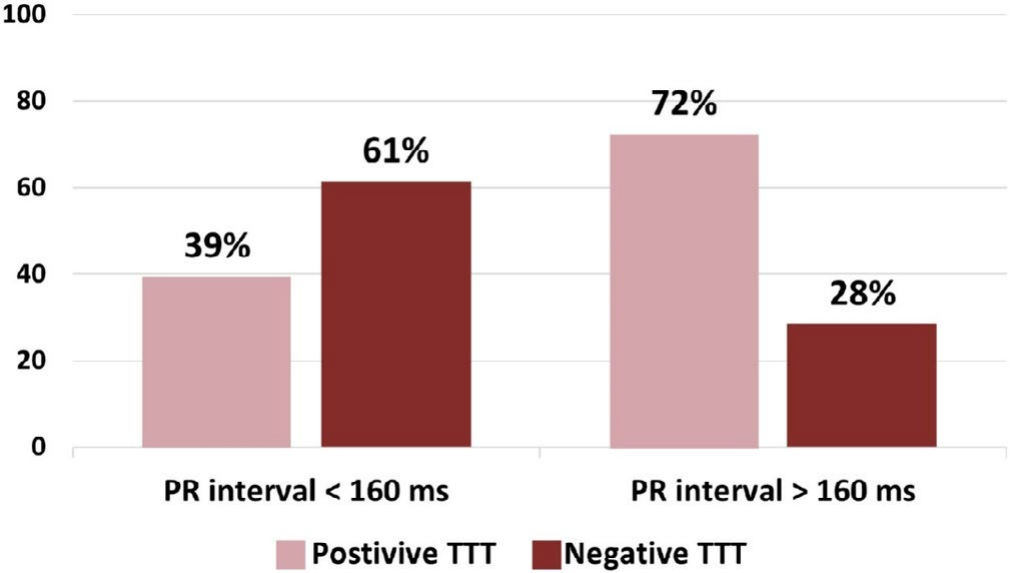
The distribution of positive and negative tilt table test based on PR interval duration.

Moreover, an increase in the PR interval was associated with a higher likelihood of a positive tilt test (OR: 1.01, 95% CI: 1.001, 1.019, *p* = 0.038). Utilizing the Youden index, we found that the optimal PR interval cutoff for predicting TTT results was 160 ms. By classifying participants based on PR interval groups, 39.33% of patients fell into the category of PR interval longer than 160 ms. Patients with PR intervals exceeding 160 ms demonstrated a higher prevalence of positive tilt tests (72%) compared to those with < 160 ms (*p* < 0.001, OR: 3.911, 95% CI: 2.143, 7.140; Figure 1).

The HRV indices including SDNN (131.60 ± 38.53 vs. 125.21 ± 14.06), SDANN (113.74 ± 35.89 vs. 112.60 ± 41.05), SDNN index (60.18 ± 23.24 vs. 53.33 ± 20.52), rMSSD (35.11 ± 16.54 vs. 30.96 ± 16.80), and pNN50 (12.56 ± 11.79 vs. 10.00 ± 11.16) were higher in TTT positive patients compared to TTT negatives. However, the differences did not reach statistical significance (Table 2).

**TABLE 2:** Comparison of autonomic nervous system activity indicators in patients with positive and negative TTT results.

	Positive TTT (Mean ± SD)	Negative TTT (Mean ± SD)	*p*
SDNN	131.60 ± 38.53	125.21 ± 14.06	0.22
SDANN	113.74 ± 35.89	112.60 ± 41.05	0.82
SDNN index	60.18 ± 23.24	53.33 ± 20.52	0.19
rMSSD	35.11 ± 16.54	30.96 ± 16.80	0.60
Pnn50	12.56 ± 11.79	10.00 ± 11.16	0.92
PR interval (ms)	164.21 ± 5.37	155.21 ± 6.65	**0.03**

Abbreviations: pNN50, percentage of successive RR intervals that differ by more than 50 ms; rMSSD, root mean square of successive differences between normal heartbeats; SDANN, standard deviation of the average NN intervals; SDNN index, mean of the standard deviations of all the NN intervals for each 5 min segment; SDNN, standard deviation of NN intervals.

## 4 Discussion

Given the high occurrence of syncope, it is crucial for physicians to differentiate between various etiologies of syncope and provide the best care. The prognosis of syncope varies from generally benign NMS cases to ventricular tachyarrhythmias with more unfavorable outcomes (Soteriades et al. 2002). Regardless of prognosis, syncope always causes anxiety and may significantly impair a person's life, necessitating prompt resolution. TTT has been implemented into the clinical evaluation of unexplained syncope as a technique to induce the vasovagal reflex in at‐risk individuals by subjecting them to controlled orthostatic stress in a secure and supervised clinical laboratory setting (Castelo et al. 2020). However, TTT requires specialized equipment, which is lacking in several emergency room and clinic environments.

### 4.1 PR Interval in Neurally Mediated Syncope

Our study proposed that the mean duration of the 24‐h PR interval recorded by AEM is a reliable predictor of TTT results, which helps differentiate NMS from other causes of syncope. The significant difference in mean PR intervals between participants with positive and negative TTT results underscores the importance of electrocardiographic parameters in assessing susceptibility to orthostatic intolerance. The longer mean PR interval observed in individuals with positive tilt test results aligns with previous studies indicating cardiac autonomic dysfunction as a contributing factor to vasovagal syncope (van Dijk, van Rossum, and Thijs 2021). Prolonged PR interval may reflect impaired cardiac autonomic regulation, which could predispose individuals to NMS and hemodynamic instability upon orthostatic challenge. Identifying a PR interval of longer than 160 ms as the optimal cut‐off for predicting TTT outcomes highlights the potential clinical utility of electrocardiographic markers in risk stratification for syncope. This cutoff provides a practical threshold for identifying individuals at increased risk of NMS, thereby facilitating targeted interventions and preventive measures. Furthermore, the demographic characteristics of the study population, including age and sex distribution, provide context for interpreting the observed associations. The wide age range and balanced sex distribution enhance the generalizability of the findings and suggest that the observed relationships between PR interval duration and tilt test outcomes are applicable across diverse patient populations.

Taking a 12‐lead ECG is highly recommended as part of the first assessment for syncope. ECG helps find any abnormal heart rhythms that may have caused the syncopal attack. Furthermore, it can show particular patterns associated with Wolff–Parkinson–White syndrome, Brugada syndrome, long‐QT syndrome, hypertrophic cardiomyopathy, or arrhythmogenic right ventricular cardiomyopathy (Shen et al. 2017b). However, the baseline ECG is presumably less useful in NMS compared to arrhythmic syncope. Nevertheless, ECG may still provide some patterns that might assist in diagnosing NMS (Țentea et al. 2021).

The ECG patterns seen in NMS are mostly associated with the cardioinhibitory reflex. These patterns are caused by varying suppression levels of the SA and AV nodes. One of the most common ECG patterns is sinus arrest, which is characterized by a gradual slowing of the sinus rhythm followed by an acceleration. There may also be instances of sinus arrest without escape after a blocked P‐wave. The depression of the AV node in NMS leads to the development of various forms of AV block, including second‐degree AV block type I or type II, 2:1 AV block, high‐grade AV block, and third‐degree AV block (Țentea et al. 2021). An investigation including 216 participants with suspected NMS who underwent the TTT as part of their diagnostic evaluation observed that the QRS voltage in frontal ECG leads was considerably lower in the TTT‐positive group of patients compared to the TTT‐negative group. The cause of this decrease in isolated QRS voltage is yet unknown. However, considering the strong link between the QRS with the lowest voltage and the LV end‐diastolic diameter, the authors proposed that isolated Very Low Voltage is likely influenced by a distinct LV geometry observed in individuals with NMS (Madias 2008).

In a study of 146 patients, Jug et al. (2021) observed PR interval shortening preceding syncope in all syncope types, regardless of gender. The shortening of the PR interval during the TTT and just before the syncope might indicate sympathetic excitation prior to the reflex bradycardia and syncope. In contrast, we evaluated the PR interval before TTT, which is the indicator of basal parasympathetic tone rather than ANS change during the table test. We concluded that a significantly longer PR interval in patients with positive TTT results might be due to the higher parasympathetic tone, which consequently makes them more susceptible to NMS. Additionally, our results demonstrated that a PR interval longer than 160 ms might be a diagnostic factor in favor of NMS. However, it is important to acknowledge that PR intervals were acquired from AEM. Further investigations are still needed to validate whether ECG‐derived PR Interval is also a predictor of TTT results.

### 4.2 Heart Rate Variability in Neurally Mediated Syncope

In several studies, HRV parameters have also been used to predict the occurrence of NMS during a head‐up tilt test (HUTT). However, the findings of various studies have been contradictory. A number of studies have shown that people diagnosed with NMS have considerably higher time domain HRV measures in comparison to other individuals (Arslan et al. 2006; Salameh et al. 2007). Arslan et al. examined the HRV parameters in 33 individuals with a typical NMS history who were scheduled for HUTT. Although not reaching statistical significance, the patients who tested positive for HUTT had higher values for NN50, pNN50, RMSSD, and SDNN indices compared to the control group. The researchers proposed that a greater level of parasympathetic activity, as shown by enhanced HRV characteristics, was linked to a positive HUTT result in individuals experiencing syncope (Arslan et al. 2006). In contrast, Zygmunt and Stanczyk (2004) discovered that the time‐domain HRV parameters were considerably lower in the tilt positive group than in the tilt negative group. Another investigation showed that cardioneuroablation significantly impacts the VV reaction, leading to the normalization of the response to TTT. This research verified that all time‐domain RV measures, including the SDNN 24‐h index, SDANN index, SDNN, and pNN50, were significantly reduced after Cardioneuroablation (Piotrowski et al. 2021).

Our study found higher HRV parameters in patients with Positive TTT. However, the difference was not statistically significant. These discrepancies might be due to the fact that the HRV does not exhibit a monotonic relationship with the parasympathetic effect (Goldberger et al. 2001). This correlation is more accurately represented by a function that exhibits an ascending limb, during which HRV increases as the parasympathetic effect increases until it reaches a plateau. As the parasympathetic effect increases beyond this threshold, HRV actually decreases. Furthermore, interindividual, sex‐related, and age‐related variations are also evident in the relationship between HRV and the parasympathetic effect (Geovanini et al. 2020). Of note, studies that observed an increase in HRV in response to baroreflex‐mediated parasympathetic stimulation likely assessed subjects primarily on the ascending limb of the correlation between HRV and parasympathetic effect.

### 4.3 Limitations

Despite these discrepancies, the collective evidence from multiple studies, including the present investigation, supports the notion that PR interval duration holds promise as an available clinically relevant predictor for TTT results and consequent NMS diagnosis. However, our study had limitations that should be acknowledged. The main limitations include the retrospective design and relatively small sample size. More studies with larger populations and further subgroup analysis based on sex and age groups are needed to reconcile conflicting findings, validate the results and our suggested cut‐off of 160 ms, and establish standardized criteria for assessing electrocardiographic parameters in the context of syncope risk stratification. Other longitudinal studies can focus on assessing the prognostic significance of PR interval duration in predicting syncope recurrence and adverse cardiovascular outcomes.

## 5 Conclusion

Our results show that a longer PR interval correlates with a positive TTT. This suggests that an AEM‐derived PR interval duration longer than 160 ms may be a valuable tool for predicting TTT results and identifying patients at higher risk of NMS. However, extending these results to conventional 12‐lead ECG needs further investigation. In that case, ECG, as one of the primary evaluations in patients with syncope, not only helps identify structural and arrhythmogenic causes of syncope but also can aids in diagnosing NMS.

## Author Contributions

M.H.N., R.N.‐J., A.A., and A.K. have contributed to designing the study, data analysis, and drafting the manuscript. S.N., S.S., H.F., A.A., M.M.M., and F.G. have contributed to the conception and critical revising of the manuscript.

## Conflicts of Interest

The authors declare no conflicts of interest.

## Data Availability

The data that support the findings of this study are available on request from the corresponding author. The data are not publicly available due to privacy or ethical restrictions.
